# Analytic Pragmatism and Universal LX Vocabulary

**DOI:** 10.1007/s11406-017-9866-4

**Published:** 2017-08-24

**Authors:** Richard Samuels, Kevin Scharp

**Affiliations:** 1 Department of Philosophy, The Ohio State University, Columbus, OH USA; 2 Department of Philosophy; Arché Philosophical Research Centre; Centre for Exoplanet Science, University of Saint Andrews, St Andrews, Fife UK

**Keywords:** Pragmatism, Conceptual analysis, Logic, Conditionals, Logical Constants, Intentionality

## Abstract

In his recent John Locke Lectures – published as *Between Saying and Doing* – Brandom extends and refines his views on the nature of language and philosophy by developing a position that he calls *Analytic Pragmatism*. Although Brandom’s project bears on an extraordinarily rich array of different philosophical issues, we focus here on the contention that certain vocabularies have a privileged status within our linguistic practices, and that when adequately understood, the practices in which these vocabularies figure can help furnish us with an account of semantic intentionality. Brandom’s claim is that such vocabularies are privileged because they are a species of what he calls *universal LX vocabulary* –roughly, vocabulary whose mastery is implicit in any linguistic practice whatsoever. We show that, contrary to Brandom’s claim, logical vocabulary per se fails to satisfy the conditions that must be met for something to count as universal LX vocabulary. Further, we show that exactly analogous considerations undermine his claim that modal vocabulary is universal LX. If our arguments are sound, then, contrary to what Brandom maintains, intentionality cannot be explicated as a “pragmatically mediated semantic phenomenon”, at any rate not of the sort that he proposes.

## Introduction

Over the past three decades or so, Robert Brandom has exerted an enormous influence on debates regarding the nature of language and philosophy. In his recent John Locke Lectures – published as *Between Saying and Doing* – Brandom further extends and refines his views on these matters by seeking to develop and defend a position that he calls *Analytic Pragmatism*. As we will see, this position combines two commitments that are exceedingly widely endorsed amongst analytic philosophers; and a careful discussion of the resulting view is of importance if for no other reason than, to date, Brandom’s position constitutes the most detailed and systematic endeavor to develop such a view.

Although Brandom’s project bears on an extraordinarily rich array of different philosophical issues, we focus here on a single central strand that runs throughout, and without which the project as a whole fails. In brief, the strand in question concerns the contention that certain vocabularies have a privileged status within our linguistic practices, and that when adequately understood, the practices in which these vocabularies figure can help furnish us with an account of semantic intentionality. More specifically, we focus on Brandom’s discussion of two vocabularies that have long figured prominently in analytic philosophy: logical vocabulary – especially the conditional – and modal vocabulary, such as that used to express necessity and possibility.^1^ Brandom’s claim is that such vocabularies are privileged because they are a species of what he calls *universal LX vocabulary* –roughly, vocabulary whose mastery is implicit in any linguistic practice *whatsoever*. On such a view, then, logical and modal vocabularies are privileged because they possess a kind of transcendental status. Further, according to Brandom, this transcendental status, at least in the case of modal vocabulary, is important to characterizing the sort of intentionality essential to thought and language.

This paper has three main aims. First, we show that logical vocabulary per se fails to satisfy the conditions that must be met for something to count as universal LX vocabulary. We focus in particular on the case of the conditional, which Brandom takes to be a paradigmatic example of logical vocabulary. If our argument goes though, then the conditional is not universal LX, and Brandom’s efforts to defend the privileged status of logic per se fail. Second, we show that exactly analogous considerations undermine the claim that modal vocabulary is universal LX. If this is so, then another of Brandom’s central claims is undermined – what he calls the *modal Kant-Sellars thesis* – the thesis that the use of ordinary, empirical vocabulary presupposes those capacities required for the introduction and deployment of modal vocabulary. Finally, we argue briefly that given our criticism of the modal Kant-Sellars thesis, Brandom’s conception of intentionality, which he articulates in the final pages of his Locke Lectures, is also untenable. If our arguments are sound, then, contrary to what Brandom maintains, intentionality cannot be explicated as a “pragmatically mediated semantic phenomenon”,^2^ at any rate not of the sort that he proposes.

Here is how we proceed. In Section 1, we provide an overview of Brandom’s Analytic Pragmatism. In Section 2, we focus on the notion of universal LX vocabulary, and both explain what it is and why is it so important to Brandom’s overarching project. In Section 3, we set out Brandom’s case for the conditional being universal LX. Then in Section 4, we present our criticisms of this case; and in Section 5, we turn our attention to modal vocabulary and argue that considerations exactly analogous to those discussed in Section 4 also undermine the modal Kant-Sellars thesis. Finally, in Section 6, we draw out the implications of the forgoing discussion for Brandom’s account of intentionality.

## Analytic Pragmatism

Brandom’s overall project – what he calls *Analytic Pragmatism* – can be thought of as a consequence of endorsing two commitments. The first is a methodological commitment, which he takes to be central to traditional analytic philosophy:

*The Analytic Project*: A central goal of philosophy ought to be to establish semantic relations between vocabularies – kinds of linguistic expressions – in order to “make sense of the meanings expressed by *one* kind of locution in terms of the meanings expressed by *another* kind of locution”.^3^ Paradigmatically, these semantic relations include translation, paraphrase, a priori entailment, and truth-making.

The history of analytic philosophy is, of course, littered with attempts to produce such analyses —including, the reduction of arithmetic to logic, the translation of psychological vocabulary into physical vocabulary, and the analysis of moral vocabulary in non-moral terms. By endorsing this commitment, then, Brandom locates his position as continuous with the project of traditional analytic philosophy.

Brandom’s second central commitment is a general thesis about the metaphysics of meaning:

*Semantic Pragmatism:* The meaning of a linguistic expression is determined by its use in a linguistic practice.

This sort of meaning pragmatism has also been prominent in the history of analytic philosophy. Though we suspect that many proponents of the analytic project have also endorsed Semantic Pragmatism, far from being a natural complement to the Analytic Project, such pragmatism poses, as Brandom notes, a serious *challenge* to it. The challenge takes a variety of forms, but the rough idea is this: If our practices determine the meanings of linguistic expressions, then it is highly implausible to suppose that expressions comprising different vocabularies – and, hence, having different meaning-determining practices – will tend to bear systematic semantic relations to each other, such as translation, reduction and paraphrase. Thus by endorsing both the Analytic Project and Semantic Pragmatism, Brandom seeks to combine a pair of commitments that seem to be in tension with each other. As a consequence, a central challenge for Brandom – and one that exerts an enormous influence on the overall shape of his Analytic Pragmatism – is to reconcile this apparent tension: to show that, contrary to appearances, one can pursue the goal of philosophical analysis at the same time as cleaving to Semantic Pragmatism.

How is this to be done? Brandom’s solution is to propose that Semantic Pragmatism requires that we rethink the fundamental semantic relations between the analyzed and analyzing vocabularies in philosophical analyses. Specifically, he proposes that if linguistic meaning is determined by use, then the relationship between distinct vocabularies should be mediated by linguistic practices.^4^ Thus, for Brandom, the way to be a pragmatist *and* to pursue the Analytic Project is to take the analyzing vocabulary to describe those practices that are sufficient to deploy the target vocabulary. If one could make good on this suggestion, then semantic pragmatists could have their analytic cake, and eat it too.

## Universal LX Vocabulary

So far we have sketched the overarching shape of Brandom’s project. But why does the notion of universal LX vocabulary come to play such a central role?

### Why Universal LX Vocabulary Matters

There are two quite different roles that the notion of universal LX vocabulary plays in Brandom’s project. The first, exemplified by its application to logic, is to justify parts of the analyzing base that everyone must presuppose in order to pursue the Analytic Project. The second, exemplified by Brandom’s discussion of modality, is to justify or vindicate the use of philosophically contested notions. In both sorts of case, however, Brandom maintains that the response should be the same. Roughly, where some vocabulary – contested or otherwise – is universal LX, we get it for free since the capacity to master that vocabulary is a necessary condition for the possibility of any language-use whatsoever. Let us consider the cases of logical vocabulary and modality in a bit more detail.

#### Logic

One way to make vivid the potential significance of the notion of universal LX vocabulary for analytic philosophy is to note, as Brandom does, that within *any* project of analysis – classical or pragmatist – logical vocabulary has a privileged status. Roughly put, no matter what the analysandum, it is taken for granted that logical vocabulary must be part of the analyzing base. If, for example, one seeks to translate psychological vocabulary into physical vocabulary, it is not merely that one uses the substantive terms of physics; one also uses – and indeed *must* use – such logical resources as conjunction, disjunction, quantifiers, and of course the conditional. But if this is so, then as Brandom notes, some justification or explanation is required for why logical vocabulary should have this privileged status. The notion of universal LX vocabulary is central to Brandom’s effort to address this challenge. Logical vocabulary is supposed to be privileged because it is a species of universal LX vocabulary, and universal LX vocabulary is supposed to be privileged.

#### Modality

At least since Locke, modal vocabulary has been a point of contention within philosophy, especially amongst those of an empiricist bent.^5^ Suspicion of alethic modality traditionally comes in at least three forms. First, there are commonplace epistemic concerns regarding how we can come to know – or justifiably believe – modal claims. Second, there are familiar metaphysical concerns about the “weirdness” of an ontology that allows for modal truths that are not merely truths of language. Finally, empiricists have long questioned the intelligibility of modal locutions —what claims to possibility or necessity might even mean. Despite these familiar concerns there has been, as Brandom notes, something of a sea change in philosophical attitudes towards modality in recent decades, in large measure due to developments in the formal semantics of modal logics. Nonetheless, Brandom argues – and we are inclined to agree – that such developments leave largely unaddressed the original philosophical challenges to modality. It is here that the notion of universal LX vocabulary is supposed to enter the picture. For if modal locutions are universal LX, then no matter what philosophical puzzlement accompanies them, they ought to be construed as holding a privileged and legitimate status. Modal vocabulary is supposed to be privileged because it is a species of universal LX vocabulary, and universal LX vocabulary is supposed to be privileged.

### What Universal LX Vocabulary is and Why It Matters

We now need to say more about Brandom’s notion of universal LX vocabulary. Here is a first pass. Universal LX vocabulary is supposed to be a privileged class of linguistic expressions in that they satisfy the following two conditions:

- The vocabulary allows one to describe practices that are necessary for having mastery for *any* vocabulary whatsoever, and
- Once one masters the described practice, one can in principle deploy the very vocabulary used to describe it.

Crudely put, Universal LX vocabulary describes the necessary conditions for mastery of any linguistic expression *whatsoever*, including universal LX vocabulary itself.

Even this rough formulation is a bit of a mouthful. But to understand clearly Brandom’s notion of universal LX vocabulary we need to explain several additional notions. First, we need to explain two notions of autonomy. The first is the notion of an autonomous *vocabulary*. To a first approximation: A vocabulary is autonomous if and only if one could use it even though one could use no other vocabulary whatsoever. Suppose, for example, that it was possible to master the language of arithmetic and nothing else. Then the language of arithmetic would be an autonomous vocabulary in the relevant sense. The second notion of autonomy – an autonomous practice – can be defined in terms of the previous notion. To a first approximation: an autonomous practice is any practice that is sufficient for mastery of an autonomous vocabulary.

As we will soon see, the paired notions of an autonomous vocabulary and an autonomous practice are important because they are required in order to specify what is it for a LX vocabulary to be *universal.* But we are getting ahead of ourselves. Before explaining what makes an LX vocabulary universal, we first need to explain what makes a vocabulary *LX*, and this in turn requires that we characterize a range of what Brandom calls *meaning-use* relations. Given the project of Analytic Pragmatism – to specify relations between vocabularies that are mediated by practices – Brandom appeals to a number of such relations:

1. *PV-Sufficiency*: Where one has mastery of any vocabulary – autonomous or otherwise – Brandom supposes that there must be some set of practices that suffices for mastery of that vocabulary. He calls this relation PV-sufficiency. Roughly: a practice is PV-sufficient for a vocabulary just in case one has mastery of the vocabulary, if one engages in that practice.
2. *PV-Necessity*: Now suppose that for some vocabulary there is a practice that one *must* engage in to count as having mastery of the vocabulary. Suppose, for example, that the capacity to identify the successor of any natural number is required for mastery of the language of arithmetic. Then this practice – identifying successors – would be PV-necessary for mastery of arithmetic vocabulary.
3. *VP-Sufficiency*: This relation between vocabularies and practices obtains when a practice can be specified in a particular vocabulary. That is, a vocabulary is VP-sufficient for a practice just in case that practice can be described using that vocabulary.^6^
4. *PP-Sufficiency*: The fourth relation that we consider here obtains between practices. If, when one engages in a particular practice, one can in principle do everything needed to engage in some other practice, then the former is PP-sufficient for the latter. For example, perhaps being able to add any three numbers between 0 and 9 is PP-sufficient for being able to find the sum of any two numbers by the usual method of addition. In characterizing PP-sufficiency, Brandom tends to express a preference for using the framework of computational theory, but there is no reason to think that this is essential to the notion.^7^
5. *PP-Necessity*: The final relation we consider is also a relation between practices. One practice is PP-necessary for another if it is impossible to engage in the second without engaging in the first. For example, the practice of asserting is PP-necessary for the practice of asserting conditionals.

With this terminology in hand, we can now provide a more explicit formulation of LX vocabulary. A vocabulary V_1_ is LX for a vocabulary V_2_ iff there exists practices P_1_ and P_2_, such that:

- P_2_ is PV-necessary for V_2_,
- P_2_ is PP-sufficient for P_1_,
- P_1_ is PV-sufficient for V_1_,
- V_1_ is VP-sufficient for P_2_.

**Fig. 1:**
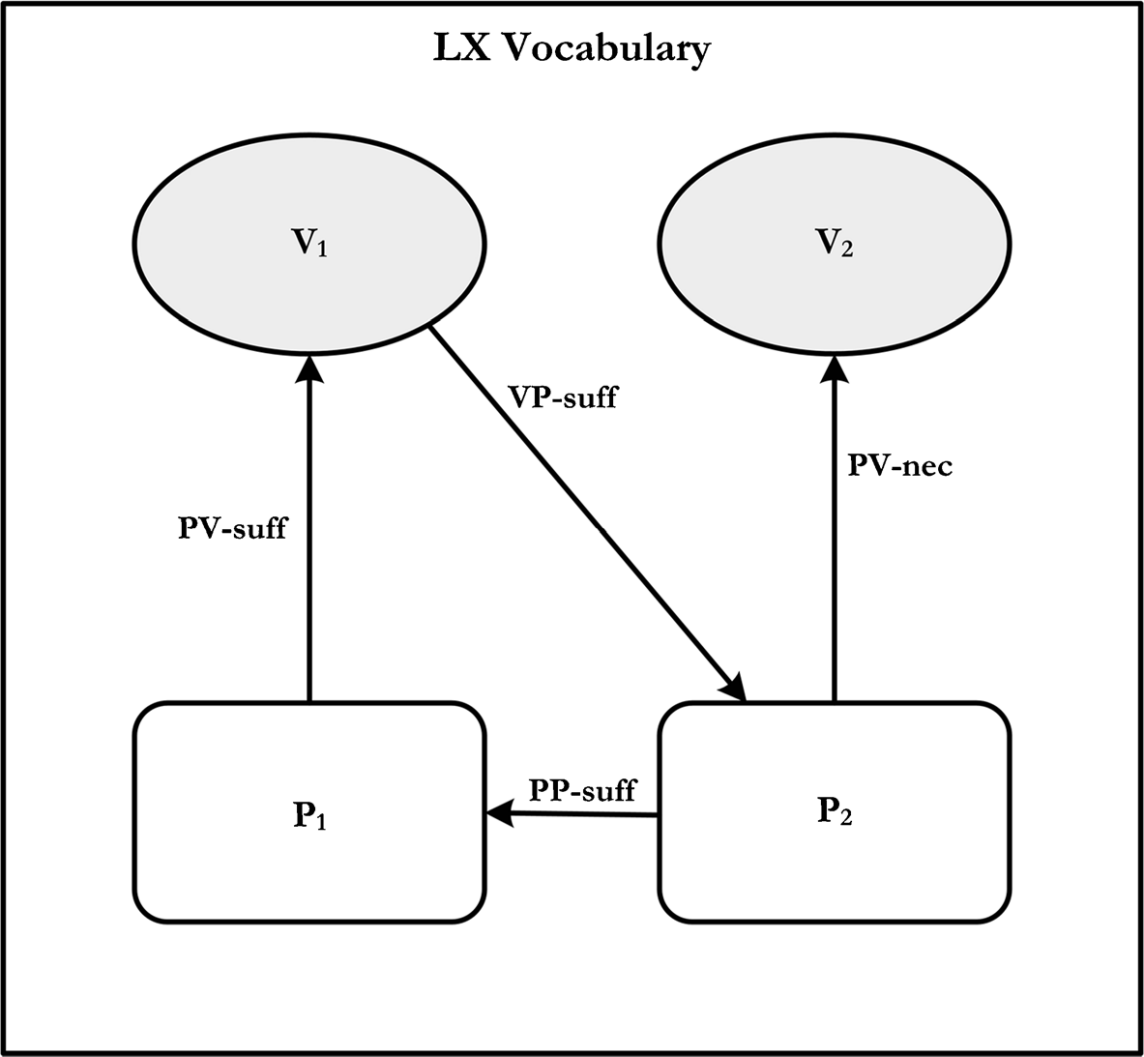
General meaning-use diagram for LX Vocabulary

In effect, what this says is that V_1_ specifies a practice P_2_ that one must be able to master in order to be a competent user of *another* vocabulary V_2_ and, moreover, the practice P_2_ incorporates everything that is required, in principle, in order to engage in a practice that the use of V_1_ depends upon –i.e., P_1_. This set of conditions can be captured, perhaps more intuitively, in what Brandom calls a meaning-use diagram (see Fig. 1).

Finally, we are in position to characterize the notion of *universal* LX vocabulary. A vocabulary is universal LX iff it is an LX vocabulary for *any* autonomous vocabulary. We can capture this in terms of an additional meaning-use relation:

(e) P_2_ is PP-necessary for *any* autonomous practice – i.e. a practice that is PV-sufficient for an autonomous vocabulary.

Thus if a vocabulary satisfies the conditions (i.e., (a)-(e)) for being universal LX, then not only are the practices it describes necessary for being a language user of any sort, but the practices so described also suffice for mastery of the universal LX vocabulary itself. As Brandom puts it for the case of logic, “anyone who can talk at all, hence can deploy any base vocabulary, can already *do* everything one needs to be able to *do* in order in principle to be able to *say* what logical vocabulary lets one *say.*”^8^ We might add that if Brandom is correct, then the same is true for modal (and normative) vocabulary as well.

## Logic as Universal LX: The Case of the Conditional

**Fig. 2:**
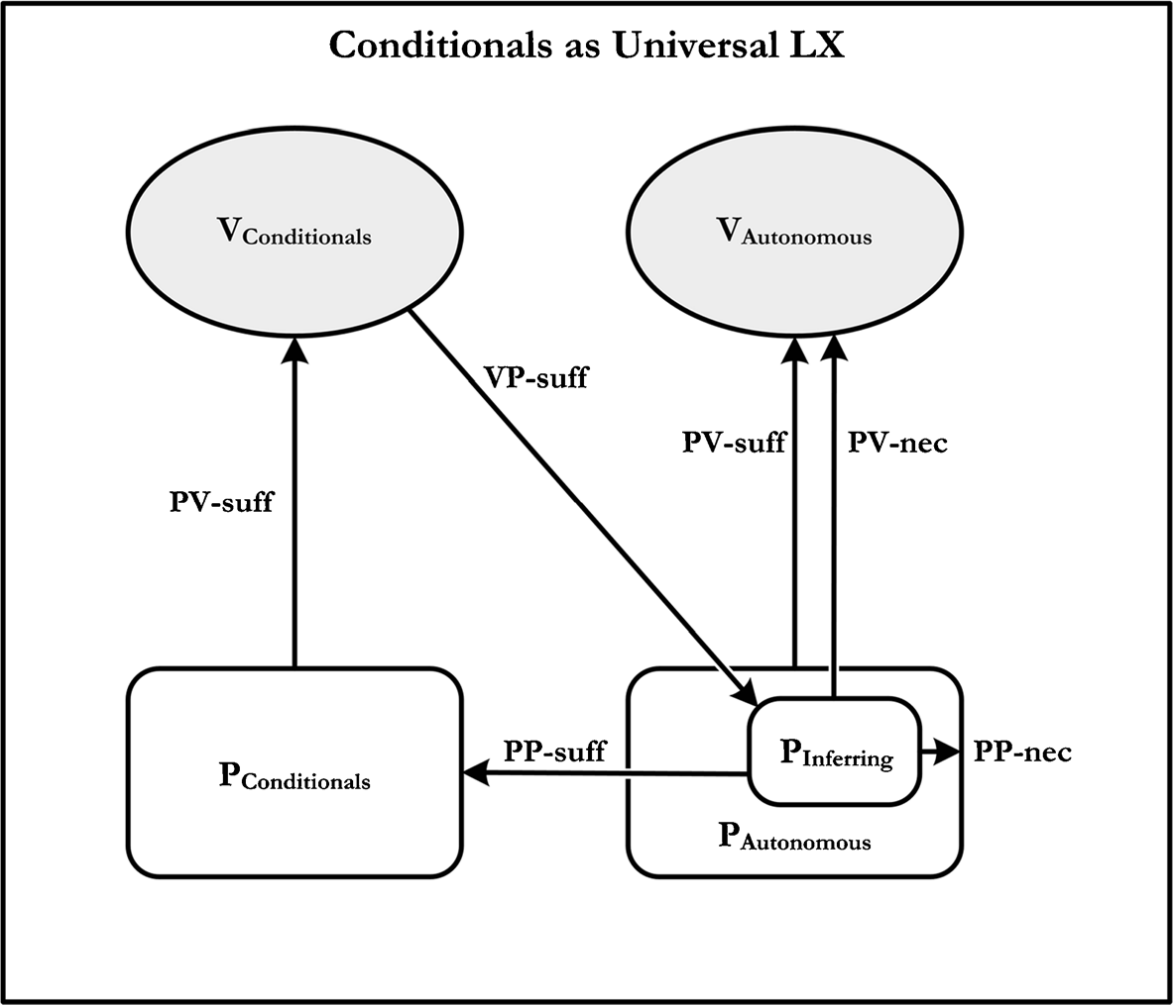
Our meaning-use diagrams differ from Brandom’s in that we list the PP-necessity relation as an arrow instead of as inclusion. See Fig. 1.9 on Brandom (2008: 28)

It is time to consider how the notion of universal LX vocabulary is supposed to apply to Brandom’s paradigmatic example of a logical expression: the conditional. In order for the conditional to be universal LX, it must be LX for any autonomous vocabulary whatsoever. So, applying the definition from Section 2.2, let us assume that V_2_ is any arbitrary autonomous vocabulary, and V_1_ is the conditional. Moreover, let us follow Brandom as characterizing P_1_ as the practice of using the conditional, and P_2_ as the practice of inferring one claim from another. In which case, again following Brandom, there are five conditions—represented by the meaning-use diagram in Fig. 2—that need to be satisfied.

*Condition 1.* According to the first condition: P_2_, the practice of inferring one claim from another, is PV-necessary for the autonomous vocabulary, V_2_. In other words, one must be able to infer one claim from another in order to count as using any vocabulary whatsoever. This is a central tenet of Brandom’s *inferentialism* on which the capacity to draw inferences is a metaphysically necessary condition on being a concept-user – hence language-user – of any sort.^9^ As Brandom puts it:

Assertions are essentially, and not just accidentally, speech acts that can play the role both of premises and as conclusions of inferences … According to this way of thinking, *inferential* practices are PP-necessary components of every autonomous discursive practice, hence PV-necessary for the deployment of every autonomous vocabulary, hence PV-necessary for the deployment of every vocabulary whatsoever.^10^

Though there are those who might wish to take issue with this claim, in what follows we grant this assumption, at least for the sake of argument.

*Condition 2.* The second condition concerns a relationship between practices. According to Brandom: P_2_, the practice of inferring, is PP-sufficient for P_1_, the practice of using the conditional. In other words, if a person can infer one claim from another, then she can, at least in principle, do everything that she needs to be able to do to count as having mastery of the conditional. Again, Brandom’s inferentialism, and the connection it assumes between asserting and inferring, is central here. According to this view, if we begin with an agent that can assert and infer, then:

…we can teach it also to produce-assertively tokenings of the new form “*if p then q.*”…The system must respond to its *assertion* of the *conditional* “if *p* then *q*” by treating the *inference* from *p* to *q* as a good one—for instance, by being disposed to endorse *q* assertionally if it is disposed to endorse *p* assertionally… In a clear sense, then, the capacity to distinguish good from bad inferences involving *non*-logical sentences is (PP-) *sufficient* for the ability to deploy conditionals involving those sentences.^11^

*Condition 3.* According to the third condition that needs to be satisfied for ‘If__, then__’ to be universal LX: P_1_, the practice of using conditionals, is PV-sufficient for mastery of V_1_, conditional vocabulary. This is true given the definitions of P_1_ and V_1_.

*Condition 4.* The fourth condition on ‘If__, then__’ being universal LX is that the vocabulary of conditionals permits us to specify adequately the practice of inferring. In other words: V_1_, the vocabulary of conditionals, is VP-sufficient for P_2_, the practice of inferring. This condition is captured by the diagonal arrow in Fig. 2. Notice that the existence of such VP-relations is crucial to the overall success of Brandom’s Analytic Pragmatism. For precisely what VP-sufficiency does is establish practice-mediated relations between pairs of vocabularies. In other words, without VP-sufficiency we would be unable to provide a practice-mediated analysis of one vocabulary in terms of another. In the present case, conditional vocabulary is taken to provide a means of specifying the practice of inference making, and thereby “make explicit” what was only implicit in the practice of drawing inferences. As Brandom puts it: “Conditionals make *explicit* something that otherwise was *implicit* in the practical sorting of non-logical inferences into good and bad.”^12^

*Condition 5:* If a vocabulary meets conditions 1–4, then it is LX. The final condition, which renders it *universal* LX, is that the practice of inferring is PP-necessary for any autonomous practice. In other words, every language-user must be proficient at inferring. Again, this is controversial; but we grant it here, at least for the sake of argument.

To summarize: Brandom maintains that conditionals are universal LX because they satisfy all of the above conditions. The burden of the next section is to show that he is mistaken.

## Conditionals are Not Universal LX

Our complaint against Brandom’s treatment of the conditional is threefold. First, we maintain that there are two quite different readings of VP-sufficiency and that there are serious problems with the claim that conditionals are, in the sense relevant to the Analytic Project, VP-sufficient for the practice of inferring. Second, even if the first objection is waived, we maintain that PP-sufficiency (i.e., Condition 2) fails. Third, even if one supplements the account so that Condition 2 holds, Condition 4 fails; and moreover it fails no matter which of the two readings of VP-sufficiency one adopts. Thus, we maintain that even if conditionals are VP-sufficient for the practice of inferring, it still follows that conditionals are not Universal LX.

### Descriptive and Expressive Notions of VP-Sufficiency

The first problem arises from the fact that Brandom’s discussion incorporates two rather different construals of VP-sufficiency: what we call the descriptive and expressive readings. The problem for Brandom is that, on the one hand, only the descriptive reading will do the work required by Analytic Pragmatism, whilst on other the other hand, conditionals can, at most, provide the basis for the expressive form of VP-sufficiency. Let us explain this in more detail.

As characterized by Brandom, the objective of Analytic Pragmatism is to provide pragmatic *analyses* —i.e., to specify, in one vocabulary, the practices that determine the content of another vocabulary. This is precisely the role that VP-sufficiency is supposed to play within the broader project. But talk of “specifying” appears, given the context, to involve *describing* a set of practices. Indeed, his paradigm cases of VP-sufficiency confirm this impression. For example, Brandom glosses what he calls the Kaplan-Stalnaker semantics for indexicals by saying the following:

[It provides a] purely non-indexical vocabulary [that] *can* serve as an adequate *pragmatic* metavocabulary for indexical vocabulary. That is, one can *say* (that is, describe), in wholly non-indexical terms, everything one needs to *do* in order to *use* indexical vocabulary. Non-indexical vocabulary is VP-sufficient to specify practices-or-abilities PV-sufficient to deploy indexical vocabulary.^13^

In short: VP-sufficiency, on this *descriptive* construal, involves the description of linguistic practices.

Let us turn to the second, *expressive* construal of VP-sufficiency. On this view, VP-sufficiency need not involve the description of practices. Instead it consists in one vocabulary making *explicit* what is only implicit in a linguistic practice. Very roughly, one makes explicit a linguistic practice or ability when one introduces an expression into the language that allows us to formulate claims about – and rationally assess – performances of the practice.^14^ To take a relatively simple example, Brandom claims in some earlier work that the negation sign renders explicit what would otherwise only be implicit in our linguistic practices — namely, treating two claims as incompatible. As he puts it:

*Negation*, as a logical connective supporting formally valid inferences, plays the same explicitating role with respect to material *incompatability* relations among judgeable (that is propositional) contents that the conditional plays with respect to the material *inferential* relations among such contents.^15^

On this expressive construal of VP-sufficiency, then, no description need occur.

The problem is that a vocabulary that only makes explicit some feature of a practice need not describe that practice. Yet precisely what pragmatic analyses seem to require is a description of the practices. One way to see the point is to consider the pragmatic “analysis” that would result from merely making explicit the practice of treating two claims as incompatible. If all that were required for VP-sufficiency was that this be made explicit, then the “analysis” would consist in one word: not! By any lights, such a result would not be an analysis worthy of the name. But it should be clear that conditionals can no more *describe* the practice of inference than the negation sign can describe the practice of treating-as-incompatible. Instead, what it more plausibly does, is make some element(s) or aspect(s) of the practice explicit. And indeed, this is what Brandom ends up emphasizing when he argues for the claim that conditionals are universal LX.^16^ Our complaint, in short, is that Brandom slides from a descriptive notion of VP-sufficiency that cannot be met by conditional vocabulary to an expressive conception of VP-sufficiency that can be played by the conditional but which is not fit for the purposes of Analytic Pragmatism.

### Inferring, Asserting and Inferring-and-Asserting

Let us waive the previous objection for the sake of argument. Still, there is another – and in our view more serious – problem for the claim that conditionals are VP-sufficient for the practice of inferring. Here the problem concerns how to understand the bottom right-hand interior box in Fig. 2. According to Brandom’s characterization of conditionals as universal LX, the practice of inferring is supposed to be PP-sufficient for the practice of using conditionals. But this cannot be right. For, in order to have mastery of conditionals, one must also be able to engage in the practice of asserting. This is a point that Brandom would himself readily endorse and for a general and rather obvious reason: a language user who is incapable of assertion would be no language user at all. But if this is so, then the practice of inferring is not PP-sufficient for the practice of using conditionals, and so conditionals (on this construal of the diagram) are not universal LX.

Now, it should be clear what is going on here. Brandom is assuming that the practices of asserting and inferring go together. As he puts it: “the beginning of wisdom” in understanding the nature of assertion “is to see that *asserting* and *inferring* are internally related practices, in the sense that each is PP-necessary for the other.”^17^ So, in order to accurately capture the meaning-use relations for conditionals, we need to augment the lower right-hand interior box in the diagram in Fig. 2 by having the conjunction of inferring *and* asserting as PP-sufficient for the practice of using conditionals. But now the problem crops up elsewhere. Even if we grant that the conjoint practice of inferring-and-asserting is PP-sufficient for the practice of using conditionals and that this practice is, in turn, PV-sufficient for the mastery of the conditional, it is simply false that the conditional locution is VP-sufficient for the practice of inferring-and-asserting. Moreover, this is so irrespective of whether one adopts a descriptive or an expressive conception of the VP-sufficiency relation.

Consider the options. On the descriptive reading, the ‘if__then__’ locution would need to describe the practice of inferring-and-asserting, which it clearly does not: any more than ‘not’ describes the practice of treating two claims as incompatible. On the alternative expressive reading, the ‘if__then__’ locution would need to make explicit the practice of inferring-and-asserting. But even if we grant that the conditional makes the practice of inferring explicit, it surely does not make inferring-and-asserting explicit. To see this, we need only reflect upon the vocabulary that *would* make asserting explicit. According to one familiar proposal, for example, the Fregean assertion stroke (├) can be used to express assertoric force. But on the face of it, the conditional, by itself, does not obviate the need for the assertion sign, any more than, say, disjunction does. When one asserts a conditional one does not thereby assert either the antecedent or the consequent. Moreover, in expressing conditionals, one does not thereby resolve the force of the utterance since conditionals can, like most other expressions, figure in assertions, suppositions, queries, commands, and so on. Thus, however we construe VP-sufficiency (whether descriptively or expressively) conditionals are not VP-sufficient for the practice of inferring-and-asserting.

In sum: The practice of inferring, by itself, is not PP-sufficient for the practice of using conditionals. Perhaps the practice of inferring-and-asserting is PP-sufficient for the practice of using conditionals. But even if this is true, conditionals are not VP-sufficient for the practice of inferring-and-asserting. Thus, either Condition 2 fails or Condition 4 fails. Either way, conditionals turn out not to be LX, much less universal LX.

## Modal Vocabulary is Not Universal LX (or Why the Modal Kant-Sellars Thesis is False)

In the preceding sections we argued at some length that Brandom’s primary example of logical vocabulary – the conditional – is not universal LX. Since the claim that logical vocabulary is universal LX is amongst the central claims of *Between Saying and Doing*, and since the conditional is the only piece of logical vocabulary that he considers at any length, we take this to pose a serious problem for his project. We now propose to challenge another central thesis of the book – what Brandom calls the *modal Kant-Sellars thesis* —roughly, the claim that modal vocabulary (e.g., ‘possible’, ‘necessary’, and ‘contingent’) is universal LX. More specifically, we maintain that it is subject to exactly analogous problems to those set out earlier.

### The Modal Kant-Sellars Thesis and Its Philosophical Significance

As noted in Section 2.1, a central goal of Brandom’s project in *Between Saying and Doing* is to argue for the thesis, which he calls the *modal Kant-Sellars thesis*, on which modal vocabulary has a privileged status akin to logical vocabulary.

Via an extensive discussion that focuses primarily on Sellars – but takes in Ryle, Kripke, and Quine along the way – Brandom fashions a characterization of the modal Kant-Sellars thesis that conforms to the pragmatic conception of meaning so central to Brandom’s overarching project. In particular, the version of the modal Kant-Sellars thesis that he endorses is formulated as the conjunction of two claims:

1. In using ordinary empirical vocabulary, one already knows how to do everything one needs to know how to do in order to introduce and deploy *modal* vocabulary.
2. The expressive role characteristic of alethic modal vocabulary is to *make explicit* semantic, conceptual connections and commitments that are already *implicit* in the use of ordinary empirical vocabulary.

**Fig. 3:**
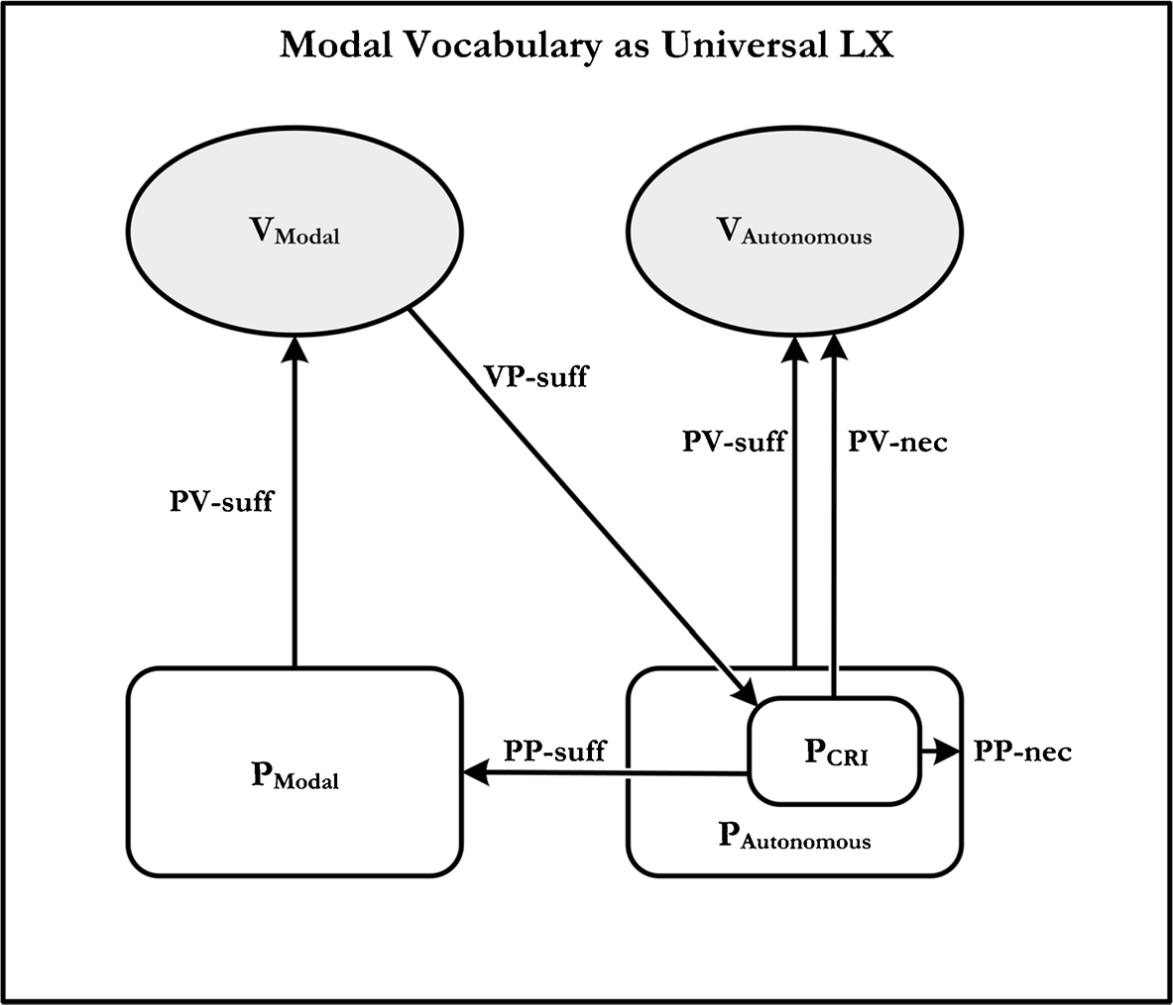
A meaning-use diagram for modal vocabulary

As Brandom observes, this formulation of the modal Kant-Sellars thesis is amenable, with minimal modification, to the sort of meaning-use analysis that we have already encountered. Claim 1 maintains that some set of practices that are PV-necessary for the use of any empirical vocabulary are PP-sufficient for practices that are PV-sufficient to deploy modal vocabulary. Claim 2 maintains that modal vocabulary makes explicit – is VP-sufficient for – those aspects of practices that are implicit in – i.e., PV-necessary for – the use of *any* empirical vocabulary whatsoever. Thus, according to Brandom’s modal Kant-Sellars thesis, modal vocabulary is universal LX. This is represented diagrammatically in Fig. 3 (‘CRI’ stands for ‘counterfactually robust inference’, to be explained below).

There is a crucial component of Brandom’s discussion of the modal Kant-Sellars thesis that we have yet to touch upon. From inspection of Fig. 3, it is clear that the thesis requires that there be *some* set of practices – corresponding to the bottom-right interior box – that satisfies three conditions:

- It is PV-necessary for using any empirical vocabulary;
- It is PP-sufficient for those practices PV-sufficient for deploying modal vocabulary; and
- Modal vocabulary is VP-sufficient for these practices.

Yet as formulated no commitment is made regarding *what* these practices are. This is a gap that Brandom fills in the course of arguing for the modal Kant-Sellars thesis.

In brief, his argumentative strategy is to justify the thesis by showing that there is a practice that satisfies the above conditions. What might it be? His answer is that it is the capacity for what he calls “counterfactually robust inference”: the capacity to associate a range of “counterfactual robustness” with each materially good inference.^18^ In other words, he maintains that mastery of any empirical vocabulary requires that one be able to distinguish the counterfactual conditions in which an inference would fail from those in which it would go through. For otherwise one would not count as possessing the relevant empirical concepts.^19^

### Why Brandom’s Modal Kant-Sellars Thesis is False

Although we do not deny the general thesis that modal notions are in some way implicit in ordinary descriptive, linguistic practices, we do reject the claim that modal vocabulary is universal LX, and with it Brandom’s specific formulation of the modal Kant-Sellars thesis. Moreover, our line of criticism is exactly analogous to the objection we leveled against the claim that the conditional is universal LX in Section 4.2.

In order to have mastery of modal locutions, one must also be able to engage in the practice of asserting —i.e. P_Modal_ in Fig. 3 must involve the ability to make assertions. This is a point that Brandom himself would readily endorse and for precisely the same reason mentioned in the case of the conditional. But if this is so, then the practice of counterfactually robust inferring (hereon CRI) is not PP-sufficient for the practice of using modal notions, and so modal vocabulary (on this construal of the diagram) is not universal LX.

Now, as with the case of the conditional, one might respond to this problem by maintaining that the capacity for asserting is PP-necessary for the capacity for CRI and vice versa. In which case, to capture the meaning-use relations for modals, we need to augment the lower interior right-hand box in the diagram by having the conjunction of CRI *and* asserting as PP-sufficient for the practice of using modal locutions. But just as before, the problem now crops up elsewhere. Even if we grant that the conjoint practice of CRI-and-asserting is PP-sufficient for the practice of using conditionals, and that this practice is, in turn, PV-sufficient for the mastery of modals, it is simply false that modal locutions are VP-sufficient for the practice of CRI-and-asserting. Moreover, this is so irrespective of whether one adopts a descriptive or an expressive conception of the VP-sufficiency relation.

Consider the options. On the descriptive reading, modal locutions would need to describe the practice of CRI-and-asserting, which they clearly do not: any more than ‘not’ describes the practice of treating two claims as incompatible. On the alternative expressive reading, modal locutions would need to make explicit the practice of CRI-and-asserting. But even if we grant that modals make the practice of CRI explicit, they surely do not make CRI-and-asserting explicit. To see this, we need only reflect again upon the vocabulary that would make asserting explicit. According to one familiar proposal, for example, the Fregean assertion stroke can be used to express assertoric force. But on the face of it, modal vocabulary, by itself does not obviate the need for the assertion sign, any more than, say, the conditional does. Thus, whichever way we construe VP-sufficiency – whether it be descriptively or expressively – modals are not VP-sufficient for the practice of CRI-and-asserting.

In sum: The practice of CRI, by itself, is not PP-sufficient for the practice of using modals. Perhaps the practice of CRI-and-asserting is PP-sufficient for the practice of using modals. But even if this is true, modals are not VP-sufficient for the practice of CRI-and-asserting. Thus, either Condition 2 fails or Condition 4 fails. Either way modals turn out not to be LX, much less universal LX.

### The Normative Kant-Sellars Thesis

We have argued that the modal Kant-Sellars thesis fails. But those familiar with *Between Saying and Doing* might wonder whether any of this applies to what Brandom calls the *normative Kant-Sellars thesis*: the claim that in order to apply ordinary, empirical, descriptive vocabulary – and hence to deploy any autonomous vocabulary whatsoever – one must already be able to do everything needed to introduce normative vocabulary.^20^ This thesis is defended in tandem with the modal version of the Kant-Sellars thesis. Moreover, Brandom appears to suppose that the two theses are strongly analogous, being answers to two variants of a single issue:

Kant read Hume's theoretical and practical philosophies as raising variants of a single question. On the side of theoretical reasoning, Hume asks what our warrant is for moving from descriptions of what *in fact* happens to characterizations of what *must* happen, and what could not happen. How, he wants to know, can we rationally justify the move from descriptions of matter-of-factual regularities to formulations of necessary laws? On the side of practical reasoning, Hume asks what our warrant is for moving from descriptions of how things are to prescriptions of how they ought to be. How, he wants to know, can we rationally justify the move from ‘is’ to ‘ought’? …

As we have seen, on the modal side, Kant's response is that Hume's predicament is not a real one. One cannot in fact fully understand the descriptive, empirical employment of ordinary determinate concepts such as cat without at least implicitly understanding also what is made explicit by the modal concepts that articulate laws. Kant mounts a corresponding line of thought on the side of *normative* or *practical* necessity. Normative concepts make explicit commitments that are implicit in any use of concepts, whether theoretically in judgment or practically in acting intentionally.^21^

In view of this apparently tight connection between the two Kant-Sellars theses one might wonder whether the normative version is subject to the same problems we level against the modal version.

**Fig. 4:**
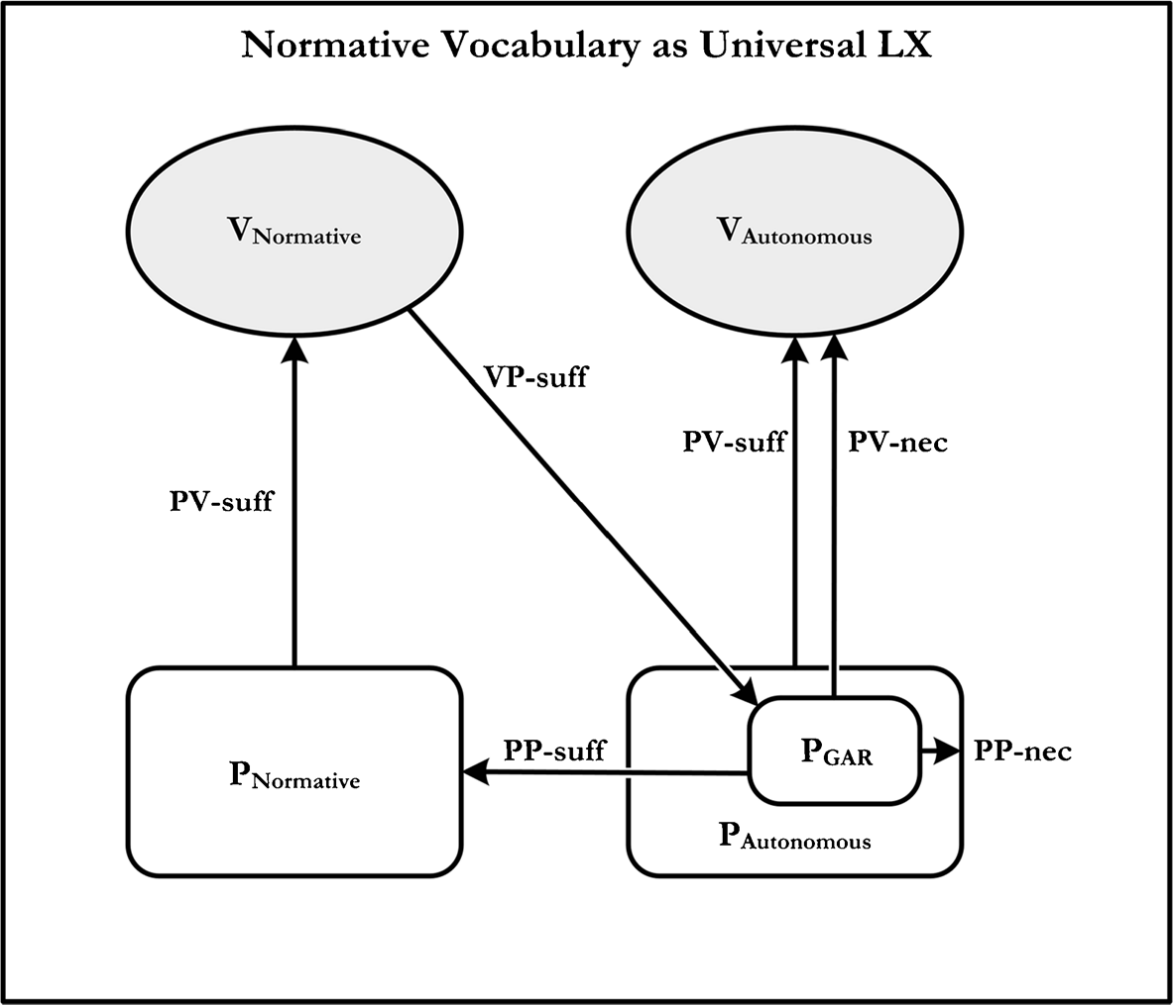
A meaning-use diagram for normative vocabulary

We suspect that the normative Kant-Sellars thesis is unscathed by the sorts of considerations raised above. In order to see this, consider the meaning-use diagram that Brandom provides for the normative Kant-Sellars thesis (‘GAR’ stands for ‘the game of giving and asking for reasons’) Fig. 4.

In order for normative vocabulary to be universal LX, it must be VP-sufficient for some practice that is, in turn, PP-sufficient for the practice of using normative vocabulary. According to Brandom the practice in question is giving and asking for reasons. But this practice is – and this is the crucial point – one that incorporates the practice of assertion. Giving a reason *just is* a kind of asserting. But if this is so, then we have no reason to doubt that it is PP-sufficient for the practice of using normative vocabulary. Thus no analog of our earlier objections can be run for the present case.^22^

## Intentionality as a Pragmatically Mediated Relation

The final central thesis of Brandom’s *Between Saying and Doing* concerns the nature of intentionality – the world-directed character of language and thought – that we call *the pragmatically mediated conception of intentionality*. In our view, this proposal is of profound importance to the overarching project of Analytic Pragmatism. Yet Brandom’s discussion is underdeveloped and, at times, frustratingly obscure. With this in mind, we propose to start with a careful and charitable reconstruction of the proposal. Having done so, we show that the arguments of the foregoing sections provide reason to reject the proposal.

### Motivation: A Persistent Criticism of Semantic Pragmatism

One way to appreciate the central motivation for – and significance of – Brandom’s pragmatically mediated conception of intentionality is to start by focusing on a persistent criticism of the semantic assumptions of Analytic Pragmatism. More or less by definition, Analytic Pragmatism is committed to *Semantic* Pragmatism: the thesis that linguistic meaning is determined by use. Moreover, the sorts of analyses that Brandom seeks to provide consist in specifying the linguistic practices on which our mastery of various vocabularies are thought to depend. But as Brandom himself readily acknowledges, there is a worry that such analyses are, in fact, not *semantic* at all; and not for any reason that has anything specific to do with the particular analyses that he advocates. Rather, the worry is quite general. Such analyses fail to be semantic – and hence to do the work required of them by Analytic Pragmatism – because they conform to the general precepts of Semantic Pragmatism, and Semantic Pragmatism is unsatisfactory as an account of semantic properties. On such a view, meaning is ultimately to be understood in terms of the linguistic practices in which we engage, not the relations that terms bear to aspects of the world. But according to many, semantics, if it is anything, is concerned with precisely such word-world relations – e.g., with representation, truth, and reference.^23^ In which case, it may seem that to the extent that Analytic Pragmatism seeks to provide *semantic* analyses, it is doomed to fail. Thus whatever else the value of pragmatic analysis, it cannot be a satisfactory extension of the project initiated by Frege, Russell and Moore.

### The Pragmatist Responds

One obvious response to the above concern is simply to deny that semantics is centrally concerned with word-world relations. Yet clearly this is not Brandom’s response. Rather, he thinks that there is something importantly right about the idea that semantic properties involve word-world relations. The challenge, then, is to explain why this is so in a manner consistent with Semantic Pragmatism. This is precisely the role that his account of intentionality is supposed to fill.

#### Step 1: The Nature of Practices

Brandom’s response can be viewed as having two main steps. The first is driven by what he takes to be a very general fact about the nature of practices, which he views as a central claim of the pragmatist tradition. Of course language use *qua* practice involves relations between subjects and the world because *in general* practices involve aspects of the world: they are *thick*.^24^ To take some simple examples, the practice of cooking cannot be adequately characterized absent such things as food items and kitchen utensils; and the practice of drinking tea cannot be adequately characterized without reference to such things as cups, saucers, and, of course, tea leaves. But such components of the practices are also aspects of the world, and thus the existence of these practices carry a commitment to subject-world relations. And what goes for cooking and tea drinking is also true of language use. *Qua* practice, it too ineliminably involves subject-world relations.

So there is a sense in which, according to Brandom, the worry with which we started is no objection at all. That is, if the concern is that Semantic Pragmatism severs the connection between word and world, then the concern is misplaced. At best it is an artifact of assuming that the relevant practices ought to be characterized “thinly”, in such a way as to avoid reference to aspects of the world. But as Brandom clearly recognizes, this does not settle the matter. The *real* problem for the semantic pragmatist is not that there are *no* relations between language-using subjects and the world. On the contrary, there are a great many such relations. Such relations are *cheap*. The real problem for semantic pragmatists is specifying the *right* relation(s) – those relevant for the purposes of characterizing the intentional character of language use; and this, according to Brandom, requires a more detailed, pragmatist account of intentionality.

#### Step 2: Combining the Accounts of Modal and Normative Vocabulary

To appreciate Brandom’s account of intentionality one needs first to make three general points clear. The first, already hinted at above, is that he views intentionality of the sort manifested by the practice of language use as a specific instance of a broader phenomenon, manifested by all human practices, which he calls *practical intentionality*. Practices quite generally, you may recall, involve relations between subjects and aspects of the world. Further, as Brandom notes, practices invariably involve what he calls practical intentionality: feedback-governed relations in which the results of earlier actions are used as guides to future, goal-directed behavior. This is true, for example, of cooking and tea drinking, but also football kicking and everything else for that matter, including language use. According to Brandom, then, the *semantic* intentionality characteristic of language use is best thought of as a specific instance of practical intentionality.^25^

The second point concerns the order of explanation one ought to adopt in explaining semantic intentionality. According to Brandom, some traditions^26^ purport to start with independent conceptions of the states of the subject that represent and of the entities so represented, and then seek to characterize the nature of the intentional relation that holds between them —to somehow “bolt” them together.^27^

Brandom maintains that such an approach is wrong-headed in that it gets the proper order of explanation exactly backwards, and thereby ignores precisely that which allows for intentionality in the first place: “the thick, essentially world-involving practices” in which we engage.^28^ In its place, Brandom proposes that should start, as traditional pragmatists have advocated, with the world-involving practices themselves; and only characterize intentionality and its relata as a kind of abstraction from these practices.^29^ Thus it is only by taking our rich, thickly characterized linguistic practices as primary and subjecting them to careful enquiry that, according to Brandom, we have any hope of understanding the general phenomenon of semantic intentionality.^30^

The final general point concerns the specific practices most central to explaining intentionality. According to Brandom, the most central are those concerned with our use of normative and modal vocabularies. Indeed his strategy is, in effect, to explain intentionality by combining the two Kant-Sellars Theses discussed in Section 5, and seeking to draw out certain consequences of their joint endorsement. In Section 6.3 we argue that this strategy does not work. But first some further unpacking is in order.

Recall: for Brandom, though an explanation of semantic intentionality requires an account of the relevant subject-world relations, such an account is to be provided by abstraction from the relevant linguistic practices. The primary task, then, is to supply an adequate account of the relevant practices; and for Brandom the two central practices are those concerning modal vocabulary and normative vocabulary, since they inform us about the *relata* of semantic intentional relations: the world and the rational subject, respectively. The practices made explicit by modal vocabulary (CRI) impose constraints on the modal structure of reality. Roughly, they reflect constraints, implicit in our thought and language, on ways that the world could be and must be. In contrast, the practices made explicit by normative vocabulary (GAR) impose constraints on rational agency. Roughly, they tell us what is it to be a rational subject – a producer and consumer of reasons. Thus for Brandom these linguistic practices, though not themselves the poles of intentional relations, are crucial to explicating the nature of intentionality, because it is by providing appropriately perspicuous characterizations of them that we are able, by abstraction, to reconstruct the nature of the relata.‬‬‬

So far so good. But an account of intentionality must do more than merely explicate those practices that concern the *relata*. Rather, one needs to explain how each of these practices are related to each other so as to yield the larger practice on which semantic intentionality depends. In effect, Brandom must address an analog of the problem that confronts more traditional approaches to intentionality of the sort that he rejects. For as we have already noted, on some approaches to the explanation of intentionality, the challenge is to specify the relevant relation between the relata: i.e., subject and world. For Brandom, the challenge is to specify the relationship between two *practices*: the world-oriented practices made explicit by modal vocabulary and the subject-oriented practices made explicit by normative vocabulary.

How is this to be done? Brandom’s answer is that the two practices intersect in what he calls *rational rectification*, which is the practice by which new commitments are integrated into one’s cognitive economy – one’s system of beliefs, desires, goals, and other intentional states. Such a practice involves the modification of one’s beliefs – often in the light of new information – so as to eliminate inconsistencies among one’s current commitments. But the capacity to do so presupposes a capacity to recognize inconsistencies, which in turn presupposes a capacity to engage in inferences that draw out the consequences of one’s current beliefs. According to Brandom, all of these cognitive capacities are aspects of the practice made explicit by use of modal vocabulary; and thus are implicit in the practice of rational rectification.

But there is more to rational rectification than this. Rational rectification requires more of us than the mere capacity to recognize inconsistency – and whatever inferential capacities this, in turn, presupposes. Among other things, this alone would be compatible with continuing to endorse inconsistent commitments. In addition to this, rational rectification requires that one take oneself to have an *obligation* to eliminate such inconsistencies – to structure one’s commitments in such a way as not to violate facts about the modal structure of the world. And according to Brandom, the practice of having one’s commitments conform to such constraints are amongst those made explicit by our use of normative vocabulary.

**Fig. 5:**
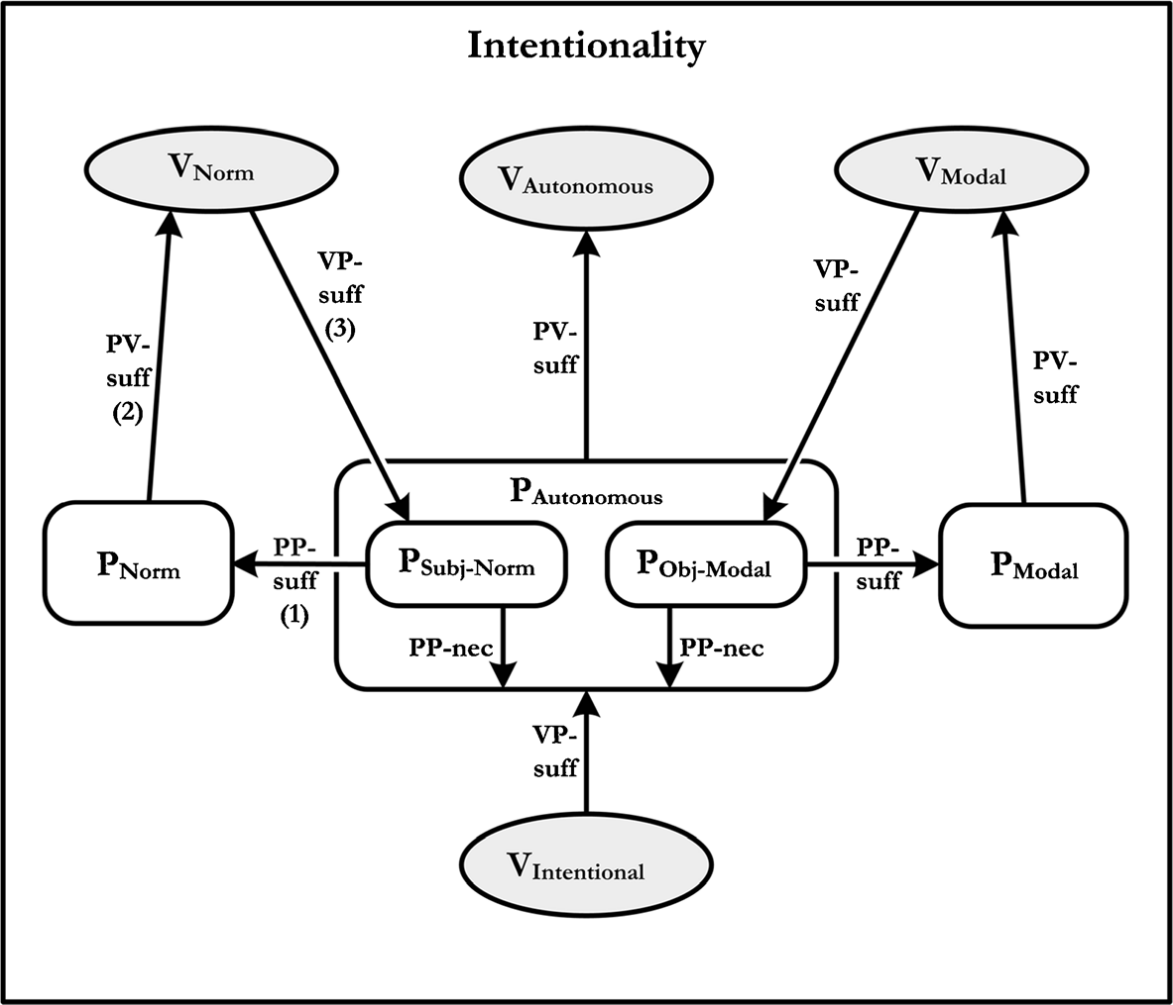
This figure has three arrows numbered for ease of reference below. See Fig. 6.1 on Brandom (2008: 183)

It is time to bring all the pieces together. What we have said so far on the topic intentionality is quite impressionistic; and indeed most of Brandom’s own discussion is similarly gestural. Nevertheless, in accord with his practice elsewhere in the book, when Brandom seeks to spell out his views precisely, he presents a meaning-use diagram. In the case of intentionality, the diagram that he uses to distill his views is reproduced as Fig. 5.

Although there is a lot going on here, we restrict ourselves to the following observations. First, the diagram aims to depict a kind of practice – indeed *any* practice of using an autonomous vocabulary – as an object for which intentional vocabulary is VP-sufficient – i.e., either made explicit by or describable by intentional vocabulary. Second, the diagram represents any autonomous discursive practice as incorporating two essential components: a practice made explicit by modal vocabulary and a practice made explicit by normative vocabulary.^31^ Thus the diagram represents mastery of such practices as necessary for being a language-user at all.

Finally, the diagram represents anyone who can engage in these two practices as being capable, in principle, of deploying the vocabularies that make these practices explicit – i.e., modal and normative vocabulary. In other words, the diagram represents the fact that Brandom’s account of intentionality – in conjunction with the earlier discussed claims regarding modal and normative vocabulary – entails the modal and normative Kant-Sellars theses. This is important for present purposes, because by tying his account of intentionality to his previous claims about modality and normativity, Brandom provides a partial explication of the nature of the practices on which intentionality depends. As we noted earlier, the real challenge for a pragmatic account of intentionality of the sort advocated by Brandom is to explicate the word-world relations that is *relevant* to intentionality; and with respect to this task, the primary significance of the Kant-Sellars theses is that they permit Brandom is specify central aspects of the relevant practices. Brandom is thus relying on his earlier discussion of modality and normativity in order to flesh out his account of intentionality.

### An Objection

In view of our earlier discussion, one problem with Brandom’s proposed account of intentionality should be clear. As we have already seen, modal vocabulary is not universal LX. So, there might be a language user that cannot already do everything needed to be able to use modal vocabulary; and if that is right, then the account of semantic intentionality, as represented in Fig. 5, *obviously* cannot be right.

How deep does this objection go? To see the significance of the objection, we need to rehearse some aspects of our earlier discussion, and introduce some features of Brandom’s theory of content. First, recall that we showed earlier that the practice of counterfactually robust inference is not PP-sufficient for the practice of using modal vocabulary. But this is just to deny that arrow 1 in Fig. 5 holds. In other words, the practice labeled P_obj-modal_ in Fig. 5 is not PP-sufficient for the practice of using modal vocabulary; and this means that Brandom has failed to characterize adequately the nature of the practice that, on his view, mediates the intentionality relation. In particular, if our arguments from Section 5 are correct, then the aspect of the practice that is supposed to explicate the “world pole” of the intentionality relation has not have been accurately characterized.

Of course, this is not the end of the matter. Following the dialectic set out in Section 5, Brandom might seek to preserve the connection between P_obj-modal_ and the practices sufficient for the use of modal vocabulary by enriching P_obj-modal_. Specifically, he might add the assumption that P_obj-modal_ is constituted, in part, by the ability to make assertions. If such a maneuver is pursued, then our earlier objection to arrow 1 fails. But now – just as before – the problem crops up elsewhere. Though arrow 2 holds more or less by definition, arrow 3 fails. That is, as argued earlier, we no longer have any reason to suppose that modal vocabulary is VP-sufficient for P_obj-modal_. In which case, modal vocabulary still fails to be universal LX, and the modal Kant-Sellars Thesis is false. As a consequence, even if one pursues this escape route, Brandom’s pragmatic theory of intentionality, at least as represented by Fig. 5, is false.

One might, however, accept our criticism and yet insist that the objection is easily met so as to preserve the spirit, though not the letter, of Brandom’s account of intentionality. Specifically, it seems eminently reasonable to ask why the account of intentionality *as such* should require that modal vocabulary be VP-sufficient for P_obj-modal_. Clearly, on anything like Brandom’s view, P_obj-modal_ should be PP-sufficient for P_modal_. Absent *this* connection Brandom would, by his own lights, have failed to explicate the “world pole” of the intentionality relation. Further, on anything like Brandom’s account, a relation of PV-sufficiency must hold between P_modal_ and V_modal_. After all, on his view, the existence of such a connection obtains more or less by definition. In contrast, it is utterly unclear why the account of intentionality requires that V_modal_ be VP-sufficient for P_obj-modal_ and, hence, that modal vocabulary be universal LX. To the extent that we concerned only with the account of intentionality, then, such a commitment seems entirely optional.

There is a sense in which we think that this response is entirely correct. Brandom’s approach to intentionality, need not – and indeed should not – demand that V_modal_ be VP-sufficient for P_obj-modal_. But we think that the assumption that it should is deeply engrained in Brandom’s views about semantics and, hence, that preserving the Brandonian approach to intentionality requires that one give up a deep-seated commitment of Brandom’s philosophical worldview, sometimes called *expressive equilibrium*. In our experience, the issue is not easily appreciated; and so we propose to creep up on the relevant assumption by first considering another assumption – *expressive completeness* – also deep-seated in Brandom’s philosophy, that does *not* suffice to mandate the commitment to arrow 3, but that does motivate the endorsement of expressive equilibrium

#### Expressive Completeness

Brandom is, of course, a semantic pragmatist, and so his theory of content takes the form of a theory of those discursive practices that are adequate to confer semantic content on the expressions involved. Expressive completeness is invoked as a condition of adequacy on such a theory. To a first approximation, expressive completeness requires that a theory of discursive practices should, in principle, be available to its practitioners. In other words, an adequate semantic theory should explain how linguistic expressions acquire their content in such a way that practitioners are, in principle, able to use the theory in order to explain the practices in which they participate. A corollary of this – one that is central to appreciating the significance of this constraint – is that such a theory will apply to *itself*. That is, an expressively complete semantic theory will correctly explain how the very linguistic expressions that comprise the theory acquire their meanings.

Why endorse such a commitment? Though we will not explore the issue in detail here, the central motivation is to avoid the sorts of self-refutation worries that notoriously confront various historically influential theories of meaning, most notably early versions of verificationism, but also Wittgenstein’s Tractarian account of meaning.^32^ Clearly, an expressively complete theory will not confront this problem since such theories are self-applicable – that is, they provide a meaning theory for the expressions in the theory itself.

Be that as it may, the assumption of expressive completeness alone fails to explain Brandom’s commitment to the VP-sufficiency of V_modal_ for P_obj-modal_. This is because the mere commitment to expressive completeness very obviously does *not* demand that *modal* expressions make explicit the practices on which *they* depend. On the face of it, all that is required is that the theory not be self-defeating – that the theory possess the resources to explain how the expressions of the theory themselves acquire their meanings. What does require VP-sufficiency, however, is the strategy that Brandom adopts in order to ensure the expressive completeness of his preferred approach to semantic theorizing – what he calls *expressive equilibrium*.

#### Expressive Equilibrium

To a first approximation, the requirement for expressive equilibrium is the requirement that the broad category of logical vocabulary – which, for Brandom, includes traditional logical connectives and quantifiers, but also normative and modal notions – makes explicit not only those norms that confer content on non-logical vocabulary, but those norms that confer content on logical vocabulary as well. To see the significance of this notion for Brandom’s semantic project we need to rehearse briefly the overall structure of the approach to semantic properties that Brandom adopts. Roughly put, he advocates a two-part approach:

1. The *basic theory:* This describes what might be called basic discursive practices – i.e. those involving no logical vocabulary. This theory will, of course, *use* logical vocabulary, but it will not explain how such expressions get their meanings.
2. The *extended theory:* This describes the extended discursive practices in which language-users engage – i.e., those involving logical vocabulary. More specifically, it does so by showing how basic discursive practices can be extended by the introduction of logical vocabulary.

Now as we have seen, the overall theory – comprised of both the basic and extended theories – needs to be expressively complete. Failing that, one risks self-defeat problems of sort that stymied verificationism. Yet clearly, participants in the *basic* practice lack the resources to state the basic theory because they posses no logical vocabulary at all. In which case, the basic theory is self-defeating. That is where the extended theory enters the picture. This theory explains how to introduce logical vocabulary – including modal and normative vocabulary – into the basic practice. So, participants in the extended practice – i.e., the practice described by the extended theory – possess the vocabulary required to state the basic theory. But now the problem recurs. In order to ensure expressive completeness of the extended theory, such practitioners must not merely have access to the basic theory, but to the extended theory as well. Otherwise, we (once more) risk regress.

Brandom’s proposed solution is that logical vocabulary is in expressive equilibrium —that it makes explicit not only those norms that confer content on non-logical vocabulary, but those norms that confer content on logical vocabulary as well. And in the parlance of *Between Saying and Doing*, this is just the claim that logical vocabulary, broadly construed to include normative and modal vocabulary, makes explicit the very practices that one needs to master in order to possess logical vocabulary itself. In short: the notion of universal LX vocabulary is a way of making more precise the demand for expressive equilibrium: a demand that Brandom thinks is central to an account of meaning that avoids self-defeat.

We are now in a position to summarize our major concerns about Brandom’s view of intentionality. First, as noted earlier, our objections to the modal Kant-Sellars thesis in Section 5 show that the view as stated is false. Second, there is no reason internal to the theory of intentionality to assume that modal vocabulary is universal LX. At most the theory of intentionality per se requires arrows 1 and 2 – not arrow 3 – because it is the first two connections that are involved in specifying the discursive practices relevant to explicating the “world pole” of the intentionality relation.

Third, arrow 3 *is* required if one seeks to endorse both the account of intentionality *and* the strategy for ensuring expressive completeness. But this is just because it is required by the theory of expressive completeness. In other words, arrow 3 comes not from the theory of intentionality but from Brandom’s strategy for achieving expressive completeness for his pragmatist theory of meaning.

Finally, once one sees the problem, it should be clear that arrow 3 is demanded by expressive equilibrium, *irrespective of what account of intentionality Brandom endorses*. That is, once one adopts expressive equilibrium as a way of attaining expressive completeness, it needs to be the case that modal vocabulary is VP-sufficient for P_obj-modal_; and more generally, that logical vocabulary, broadly construed, is VP-sufficient for those practices needed to master exactly that logical vocabulary. What our arguments in the earlier sections of this paper show is that this strategy cannot succeed.

## Footnotes

1 Following Brandom, we are interested here in aleithic modality as opposed to epistemic modality.

2 Brandom (2008: 189).

3 Brandom (2008: 1).

4 Terminological note: Brandom tends variously to use the expressions ‘practice’, ‘activity’, ‘ability’, ‘exercise of a practice’, and ‘practice-or-ability’ more-or-less interchangeably; see Brandom (2008: 9). For simplicity and ease of exposition we just use the term ‘practice’.

5 The same is, of course, true of normative vocabulary —a topic in which Brandom also invests much time in his Locke lectures.

6 We discuss this relation in far greater detail in Section 4.1.

7 See Brandom (2008: ch. 2).

8 Brandom (2008: 52-53).

9 See Brandom (1994).

10 Brandom (2008: 42-43).

11 Brandom (2008: 45).

12 Brandom (2008: 46).

13 Brandom (2008: 56).

14 Brandom’s notion of making explicit is a notoriously hard one to articulate precisely. The present way of formulating the idea might sound as if making a practice explicit consists in producing a name for that practice. For reasons that we need not go into here, however, it is quite clear that this is not Brandom’s intention. Moreover, it is clearly false that logical expressions, such as negation, are names for practices.

15 Brandom (1994: 115).

16 Brandom (2008: 44-47).

17 Brandom (2008: 42).

18 Brandom (2008: 103).

19 Brandom illustrates the point with the following example: “One grasps the claim ‘the lioness is hungry’ only insofar as one takes it to have various consequences (which *would* be true if it *were* true) and to rule out some others (which *would not* be true if it *were* true). And it is not intelligible that one should endorse as materially good an inference involving it, such as the inference from ‘the lioness is hungry’ to ‘nearby prey animals visible to and accessible by the lioness are in danger of being eaten,’ but be disposed to make no distinction at all between collateral premises that would, and those that would not, if true infirm the inference.” (p. 105)

20 Anyone familiar with Brandom’s earlier work, *Making It Explicit*, should recognize that such a thesis about normative vocabulary is central to Brandom’s philosophical position.

21 Brandom (2008: 109-110).

22 This is not, of course, to deny that some other objection might apply. But we put such issues to aside here.

23 See Rosen (2001) for example. As David Lewis once wrote in this spirit, “semantics with no treatment of truth conditions is not semantics” (Lewis 1983: 190).

24 Brandom labels this principle P3; Brandom (2008: 178).

25 Brandom labels this claim P4; Brandom (2008: 179).

26 Though he does not explicitly mention any philosophical theories that cleave this methodology, we suspect that he has in mind, amongst other things, causal theories of content, such as those advocated by Fodor and Dretske; see Dretske 1981) and Fodor (1992).

27 Brandom (2008: 179).

28 Brandom (2008: 180).

29 Historical note: This sort of view was characteristic of the views of pragmatists, such as Dewey and James, but similar views can also be found in Russell (1945: 812) among others.

30 Superficial appearances not withstanding, Brandom is not claiming that there could be no objective world without the existence of our linguistic practices. His purpose is not to advocate some kind of linguistic idealism, but instead to defend an approach to the study of intentionality.

31 Brandom uses the term ‘discursive’ as a general term for any activity in which only rational entities can engage.

32 Wittgenstein (1922).
